# Robust genotyping tool for autosomal recessive type of limb-girdle muscular dystrophies

**DOI:** 10.1186/s12891-016-1058-z

**Published:** 2016-05-04

**Authors:** Inna Inashkina, Eriks Jankevics, Janis Stavusis, Inta Vasiljeva, Kristine Viksne, Ieva Micule, Jurgis Strautmanis, Maruta S. Naudina, Loreta Cimbalistiene, Vaidutis Kucinskas, Astrida Krumina, Algirdas Utkus, Birute Burnyte, Ausra Matuleviciene, Baiba Lace

**Affiliations:** Biomedical Research and Study Centre, Ratsupites str. 1, k-1, LV-1067 Riga, Latvia; Department of Human and Medical Genetics, Faculty of Medicine, Vilnius University, Vilnius, Lithuania; Centre for Medical Genetics, Vilnius University Hospital Santariškių Klinikos, Santariškių str. 2, LT-08661 Vilnius, Lithuania; Laval University, Quebec, Canada; Centre hospitalier universitaire de Québec, 2705, boulevard Laurier, Québec, Québec G1V 4G2 Canada

**Keywords:** Limb-girdle muscular dystrophies, Illumina VeraCode GoldenGate, Calpain 3 c.550delA, Fukutin related protein

## Abstract

**Background:**

Limb-girdle muscular dystrophies are characterized by predominant involvement of the shoulder and pelvic girdle and trunk muscle groups. Currently, there are 31 genes implicated in the different forms of limb-girdle muscular dystrophies, which exhibit similar phenotypes and clinical overlap; therefore, advanced molecular techniques are required to achieve differential diagnosis.

**Methods:**

We investigated 26 patients from Latvia and 34 patients from Lithuania with clinical symptoms of limb-girdle muscular dystrophies, along with 565 healthy unrelated controls from general and ethnic populations using our developed test kit based on the Illumina VeraCode GoldenGate genotyping platform, Ion AmpliSeq Inherited Disease Panel and direct sequencing of mutations in calpain 3 (*CAPN3*), anoctamin 5 (*ANO5*) and fukutin related protein (*FKRP*) genes.

**Results:**

Analysis revealed a homozygous *CAPN3* c.550delA mutation in eight patients and three heterozygous variants in controls: dysferlin (*DYSF*) c.5028delG, *CAPN3* c.2288A > G, and *FKRP* c.135C > T. Additionally, three mutations within *FKRP* gene were found: homozygous c.826C > A, and two compound – c.826C > A/c.404_405insT and c.826C > A/c.204_206delCTC mutations, and one mutation within *CLCN1* gene – c.2680C > T p.Arg894Ter. *ANO5* c.191dupA was not present.

**Conclusions:**

Genetic diagnosis was possible in 12 of 60 patients (20 %). The allele frequency of *CAPN3* gene mutation c.550delA in Latvia is 0.0016 and in Lithuania - 0.0029. The allele frequencies of *CAPN3* gene mutation c.2288A > G and *DYSF* gene mutation c.4872delG are 0.003.

**Electronic supplementary material:**

The online version of this article (doi:10.1186/s12891-016-1058-z) contains supplementary material, which is available to authorized users.

## Background

Limb-girdle muscular dystrophies (LGMD) are a heterogeneous group of diseases that predominantly affect pelvic and shoulder girdle muscle groups. LGMD is a rare disorder, and the different forms of LGMD range in prevalence from 1 in 14,500 to 1 in 123,000, depending on the population [1, 2]. The autosomal recessive form of LGMD (LGMD type 2; LGMD-2) is more common, with 23 causal genes and chromosomal loci identified to date [3–7]. The rarer autosomal dominant (LGMD type 1; LGMD-1) form accounts for only 10 % of all cases and has eight causative genes and chromosomal loci currently identified [6, 8, 9]. These genes encode proteins involved in many different aspects of muscle cell biology. Diagnosing of specific types of LGMD is a challenging process due to the many genes involved, including unknown genes within the identified chromosomal loci. Although common mutations have been identified for some causative genes (for example, calpain 3 (*CAPN3*) c.550delA or fukutin related protein (*FKRP*) c.826C > A), a lot of additional mutations were found in different populations. Confirming the diagnosis of LGMD, in addition to subtyping, is usually done using molecular techniques. Directly sequencing the coding regions of the gene is the most common method, but remains often insufficient, due to the size and number of genes involved. Hence, the advancement of LGMD diagnostics was achieved through next generation sequencing of the genome or exome, or through targeted exome capture applications. Furthermore, several commercially available kits include loci for the diagnosis of neuromuscular disorders. The advantage of next generation sequencing lies in the capability of analyzing whole exomes at a reasonable cost without invasive muscle biopsies. Limitations of this method are related to insufficient exon coverage, leading to a potential lack of a representative examination of all exons [10].

The aim of this study was to investigate LGMD type 2 mutation spectrum and allelic frequency in Latvian and Lithuanian populations.

## Methods

### Patients

We investigated 26 patients from Latvia and 34 patients from Lithuania with clinical symptoms of LGMD. After mutation identification, segregation analysis of the family was performed.

Patients were recruited from the Neurology department of Children’s University Hospital, from the outpatient clinic for Neurological diseases at Pauls Stradins Clinical University Hospital (Riga, Latvia), and from the Center for Medical Genetics, Vilnius University Hospital Santariskiu Clinics (Vilnius, Lithuania).

The Latvian patient sample consisted of 26 unrelated individuals with predominant pelvic and shoulder girdle muscle weakness and elevated creatine kinase (CK) values. Patients with electromyography data demonstrating myopathy or myogenic impairment were included in the study. A muscle biopsy was obtained from one patient; however, analysis of this was inconclusive regarding their LGMD subtype. The mean patient age at the onset of symptoms was 14 years (range: 1 month (accidental finding without clinical symptoms until 8 years) to 53 years), and the male:female ratio was 1.4:1.

The Lithuanian patient sample consisted of 34 unrelated patients with predominant pelvic and shoulder girdle muscle weakness and elevated CK values. Mean patient age at presentation was 23 years (range: 4–60 years) and the male:female ratio was 1:2.4.

The data collection was performed with permission from the Central Medical Ethics Committee of Latvia. All participants or their parents (in case of patient’s age under 14 in Latvia and under 18 in Lithuania) signed an informed consent forms to participate and publish. Complete patient and family histories were taken at the clinic, and patients were asked about their muscle strength and performance before the onset of symptoms. Patients were interviewed and examined by a neurologist and a clinical geneticist experienced in neuromuscular disorders.

Healthy, unrelated individuals (*n* = 204) randomly selected from the Latvian Genome Database were used as controls representing the general population. When necessary, we supplemented the study with 101 samples, for total number of 394. Additionally, 175 DNA samples from Lithuania and 186 samples from Latvia were used as controls for ethnic groups. They were recruited, if their ancestors from the last three generations could be traced back archaeologically and ethno-linguistically to distinct regions of Latvia and Lithuania [11, 12].

### Microarray genotyping

We selected the highly parallel genome-wide VeraCode GoldenGate system (Illumina, San Diego, CA, USA) as the platform for mutation screening [13]. The main resources used for manually selecting mutations, previously published as causative for LGMD-2, were the open access databases [14–17] as well as publications. Mutations were selected based on their pathogenicity, location within exons or splice sites, allele frequency, and the population analyzed. In addition, the mutations had to satisfy the following criteria to comply with the VeraCode GoldenGate system: they had to be single-nucleotide mutations (non-synonymous single nucleotide polymorphisms (SNPs) or insertions/deletions (indel)); diallelic (rather than triallelic or tetra-allelic) to ensure allele specific hybridization, extension, and ligation; and be >60 base pairs away from any other mutations on the array to prevent overlapping oligonucleotides during hybridization. All LGMD patients and general population controls were genotyped for the selected mutation using a customized VeraCode GoldenGate system on a BeadXpress reader (Illumina). Reactions and quality controls were carried out under the standard conditions. To ensure quality control and to evaluate the intra-subject concordance rate, 48 duplicate samples were processed in one genotyping run, instead of 96 single samples.

The presence of the identified mutations was confirmed by direct sequencing performed using standard PCR conditions. The oligonucleotide sequences are available upon request.

### *CAPN3* and anoctamin 5 (*ANO5*) mutation detection

The mutations in *CAPN3* and *ANO5* genes (c.550delA and c.191dupA, respectively) in the Latvian and Lithuanian populations were identified by direct sequencing performed using standard polymerase chain reaction (PCR) conditions (t_annealing_ 56 °C) with the oligonucleotides *CAPN3* F1 5’ TCTCAAAAAGCACCCAGTCC 3’ and *CAPN3* R1 5’ GCCTCTTCCTGTGAGTAGG 3’, and *ANO5* F1 5’ TGGTATGTTTGCAGCATTTTTG 3’and *ANO5* R1 5’ CATACCTCAAATTTTGGATGTTAC 3’.

The frequencies of mutations in the ethnic and general populations were calculated using the Hardy-Weinberg equation under the assumption of the absence of evolutionary forces.

### *FKRP* gene sequencing

A 1.7 kb fragment containing *FKRP* exon 4 and entire coding sequence was amplified using previously published primers [18] and 1.25 Units of Long PCR Enzyme Mix (ThermoScientific, Lithuania). PCR conditions were as follows: 45 cycles, annealing temperature 52 °C, extension temperature 68 °C, and DMSO concentration 4 %.

The exon 4 end exon-intron boundaries were analyzed by direct sequencing performed using published [18] and following oligonucleotides: *FKRP* F1 5’AGATGCTGTGGTGCTCCTG 3’, *FKRP* R1 5’TCCAAGTAGATGCCGAGGTC 3’, *FKRP* F2 5’AGCTGCTGGACTTGACCTTC 3’, and *FKRP* F2 5’CCCACGTCCTCCAAGTAG 3’.

An automatic genetic analyzer (ABI PRISM 3130xl, Applied Biosystems, Life Technologies, Carlsbad, CA, USA) was used in all cases.

### Next generation sequencing using AmpliSeq Inherited Disease Panel

Life Technologies Ion Torrents targeted gene enrichment and sequencing technology AmpliSeq was performed according to a protocol provided by the manufacturer on four patient samples.

The commercially available AmpliSeq Inherited Disease Panel (Life Technologies) that covers 325 gene exons and intron-exon boundaries of most common inherited diseases, including genes of LGMD and other muscle dystrophies, was used for this approach [19]. Standard DNA extraction was followed by PCR amplification using three primer pools included in the panel. The amplicon mixes were combined and treated with FuPa reagent (Life Technologies) to partially digest primer sequences and phosphorylate the amplicons, which were then ligated to multiplexing barcodes (Life Technologies). Library was then purified using Beckman Coulter (Nyon, Switzerland) AMPure XP magnetic beads and amplified using PCR assay provided, followed by another purification. Massive parallel sequencing of library created was done using Ion Torrent PGM device.

## Results

A total of 264 DNA samples, 26 from Latvian patients, 34 from Lithuanian patients, and 204 healthy controls, were analyzed by a custom microarray for the selected loci.

We initially selected 209 sequence’s variants based on the criteria mentioned above. Of these, 121 were mutations in the *CAPN3* gene, 50 were mutations in dysferlin (*DYSF*), 28 were mutations in sarcoglycan alpha (*SGCA*), sarcoglycan beta (*SGCB*), sarcoglycan delta (*SGCD*), and sarcoglycan gamma (*SGCG*), two were gender controls (amelogenine, X-linked/ amelogenine, Y-linked), and eight were control polymorphisms. This initial number of mutations was reduced considerably due to designs resulting in critical failure scores, as calculated by the Assay Design Tool. To increase the overall performance and sensitivity of the assay, we had to decrease the investigated variants to a final total of 96.

Data cleaning was completed as recommended by the manufacturer resulting in exclusion of 23 samples.

In addition, we did a “paired sample” test in the final quality control step to compare the call frequency values of the duplicate samples, which led to the classification of 13 additional mutations as false positives and decrease the analyzed sample size to 185 (80 %). Additional file 1: Table S1 contains information about quality the parameters. A complete list of the Group 1 mutations, which have the best test performance (*n* = 20) is provided in Additional file 1: Table S2A. The additional mutations with fluctuating results included in the test (*n* = 57) form Group 2 and are provided in Additional file 1: Table S2B. Excluded mutations are provided in Additional file 1: Table S2C. After cleaning the data, we identified four different mutations in our cohort of patients and controls. Our analysis revealed the c.550delA homozygous mutation in *CAPN3* in eight Lithuanian cases (24 %). Controls from the Latvian population did not have *CAPN3* c.550delA, but had two causative mutations: *DYSF* c.4872delG and *CAPN3* c.2288A > C, and one benign SNP *FKRP* c.135C > T (one heterozygous allele each; see Table 1). These three variants were not detected in patients. Re-sequencing of these mutations revealed one false negative case within the data from the Illumina VeraCode GoldenGate assay. Thus, the sensitivity of the test was 89 % (95 % confidence interval (CI): 0.54–0.99), and the specificity was 98 % (95 % CI:0.89–0.99).

**Table 1:** Mutations identified with LGMD-2 diagnostic test kit

Name	Gene	Mutation	Frequency in Control group	Frequency in the group of patients with NMD	Known mutation frequency
CAPN3_00010 (rs80338800)	*CAPN3*	c.550delA	0 (0.001^a^)	0.13	NA
CAPN3_00119	*CAPN3*	c.2288A>C	0.003	0	NA
DYSF_00178	*DYSF*	c.4872delG	0.003	0	NA
rs2287717	*FKRP*	c.135C>T	0.003	0	0.14

^a^frequency estimated for enlarged control group (with additional 101)

*NA*, data not available

*NMD,* neuromuscular disorders

Because no controls had the *CAPN3* c.550delA mutation, it was not possible to estimate the frequency of this allele in the populations. For estimating its allele frequency in the Latvian population, an additional 101 individuals were selected from the Latvian Genome Database. One heterozygous carrier was found, and they had no symptoms of neuromuscular disease. Thus, the allele frequency of c.550delA within the Latvian population is estimated to be 0.0016 (0.16 %). However, the situation was different in the Lithuanian samples, in which the c.550delA allele was found in one of 175 samples, making the calculated allele frequency 0.0029 (0.29 %). Using the Hardy-Weinberg equation, the estimated number of persons homozygous for *CAPN3* c.550delA in the Latvian population is 0.3 per 100,000 persons, and in the Lithuanian population, it is 0.8 per 100,000. Other three variants *DYSF* c.4872delG, *CAPN3* c.2288A > C, and *FKRP* c.135C > T had an allele frequency of 0.3 % in Latvian population, and the calculated number of homozygous persons was 1 in 166,000 for each of them.

Data from patients who had been diagnosed with LGMD2A using the GoldenGate VeraCode assay, were summarized. Information about these patients’ clinical symptoms is in Table 2. To further ensure our results, we performed mutation analysis in the patients’ families, and confirmed mutations in two affected siblings. The segregation analysis was concordant with the clinical picture and the presence of the mutation.

**Table 2:** Description of patients with identified mutations

Gene	Patient ID	Mutation	Symptoms
*FKRP*		c.826C>A/c.826C>A	Female, disease onset at age 11, shoulder girdle muscle weakness and atrophy, pseudohypertrophy of calves, hyperlordosis, respiratory difficulty, CK level (U/l) 700-1500
*FKRP*		c.826C>A/c.404_405insT	Male, disease onset at age 5 with rhabdomyolysis, hypertrophic cardiomyopathy, hypertrophy of calves, mild proximal weakness, Gowers’ symptom negative, CK level (U/l) 32,000
*FKRP*		c.826C>A/c.204_206delCTC	Female, age 8, slight proximal muscle weakness, CK level (U/l) 700
*CLCN1*		c.2680C>T/N	Female, disease onset at age 4, severe proximal muscle weakness, Gowers’ symptom positive, myotonia by EMG, CK level (U/l) 13,059
*CAPN3*	D06	c.550delA/c.550delA	Female, disease onset at age 4, tip toe walking, proximal muscle weakness and atrophy, scoliosis at age 10, ankle and elbow contractures in teen years, significantly reduced physical activity at age 19, still walking after 34 years from onset, CK level (U/l) 4000 at present.
*CAPN3*	D07	c.550delA/c.550delA	Female, disease onset at pre-school age, walking and gait disorders, proximal muscle weakness and atrophy, CK level (U/l) 9001
*CAPN3*	D08	c.550delA/c.550delA	Female, disease onset at age 11, tip toe walking, waddling gait, frequent falling and inability to run, scapular winging, hypertrophy of calves, still walking after 16 years from onset, CK level (U/l) 5722
*CAPN3*	D09	c.550delA/c.550delA	Female, disease onset at age 7 with history of frequent falling and inability to run, short stature, hypertrophy of calves, proximal muscle weakness, scapular winging, walking with assistance at the age 17, CK level (U/l) 4000
*CAPN3*	D10	c.550delA/c.550delA	Female, disease onset at age 6, muscle weakness and atrophy in the lower extremities with difficulty running, climbing stairs, and frequent falls, waddling gait, scoliosis, dependent on a wheelchair at age 17, CK level (U/l) 18,826
*CAPN3*	D16	c.550delA/c.550delA	Male, disease onset at age 14, *pes equinovarus* from birth, waddling gait, fatigue in his arms progressively worsened to symmetric weakness in hip girdle, scapular winging, walking with assistance at age 29, dependent on a wheelchair at age 32, CK level (U/l) 6370
*CAPN3*	D23	c.550delA/c.550delA	Female, disease onset at age 7, proximal muscle weakness, still walking after 34 years from onset, CK level (U/l) 7487
*CAPN3*	D24	c.550delA/c.550delA	Male, disease onset at age 3, tip toe walking, proximal muscle weakness, hypertrophy of calves, significant loss of physical activity at age 14, CK level (U/l) 854

*CK*, creatine kinase, *EMG*, electromyography

As a recent and population-specific finding, mutation c.191dupA of the *ANO5* gene was also analyzed in patients and ethnic control groups because of its reported frequency in North European populations [20]. Analysis did not reveal the variant in any of the samples, resulting in an allele frequency of zero in Latvia and Lithuania.

Since none of selected mutations were found within Latvian patients’ samples, we performed analysis by direct sequencing of entire coding region and 4th exon-intron boundaries of *FKRP* gene for 23 Latvian patients. 26 Lithuanian patients, who had not been diagnosed with LGMD2A, were also included in this analysis. Our analysis revealed one c.286C > A homozygous mutation in Lithuanian patient and two compound heterozygous mutations (one in each of Lithuanian and Latvian case) within the coding region of *FKRP* gene. The first is *FKRP* c.826C > A/c.404_405insT mutation, unknown *cis* or *trans* position, another one is *FKRP* c.826C > A/c.204_206delCTC in *trans* position that is confirmed by segregation analysis, carried out in the patient’s parents samples. Information about patients’ clinical symptoms is summarized in Table 2.

Additionally, next generation sequencing using AmpliSeq Inherited Disease Panel was performed on four patient samples. These patients had no diagnosis found by other methods described. One heterozygous mutation within chloride voltage-gated channel 1 (*CLCN1*) gene – c. 2680C > T p.894R > X was identified. Patients’ clinical features are described in Table 2.

## Discussion

This study, using the LGMD-test kit, revealed a homozygous *CAPN3* c.550delA mutation in eight Lithuanian patients and three variants (one heterozygous allele each) in controls: *DYSF* c.5028delG, *CAPN3* c.2288A > G (both were reported as pathogenic), and *FKRP* c.135C > T (benign polymorphism). These three variants were not detected in patients.

The mutation *CAPN3* c.550delA is one of the most frequent among patients suffering from LGMD-2. It has been identified in many countries, including France, Greece, Italy, the Netherlands, Germany, and the United Kingdom, but was relatively infrequent in these populations [21, 22]. Relatively high frequencies (40–70 % of LGMD2A cases) of *CAPN3 c.*550delA have been observed and reported in Turkey, Bulgaria, Croatia, the Czech Republic, Poland, and Russia [23–29]. Canki-Klain et al. suggested that the *CAPN3* c.550delA mutation originated in the Eastern Mediterranean, probably spreading widely across Europe, and single haplotype analysis confirms this hypothesis [27].

Our data suggest that *CAPN3* c.550delA is also the most frequent mutation in LGMD patients in Lithuanian populations. The calculated frequency of homozygous persons is 0.8 per 100,000 people, and in Latvia calculated frequency is 0.3 in 100,000. The marked difference observed between allele frequencies in Lithuanian and Latvian populations remains unexplained. We speculate that the disease gradient falls rapidly towards the North and East of Europe.

The *CAPN3* gene mutation c.2288A > G and *DYSF* gene mutation c.5028delG (currently c.4872delG) carrier frequency is 1 in 204 people.

We did not find common mutations in *ANO5*, *SGCA*, *SGCB*, *SGCD*, or *SGCG*. It is possible that these common mutations have distinct founder effects and are sparsely scattered in Northeast Europe.

Additionally, we found three different mutations within *FKRP* gene: c.826C > A, c.404_405insT, and c.204_206delCTC and one mutation in *CLCN1* gene – c.2680C > T.

Currently, the LGMD-2 diagnosis toolkit is not vital for diagnostics; nevertheless, the information about the mutation spectrum we acquired in this study is important for construction a gene panel for LGMD diagnostics and gives insight into the mutation spectrum of this heterogeneous disease.

## Conclusions

The LGMD-2 test kit was introduced as a rapid and low cost screening tool for improvement of the healthcare of the neuromuscular disease patients. Genetic diagnosis was achieved in 12 of 60 patients (20 %). The allele frequency of *CAPN3* gene mutation c.550delA in Latvia is 0.0016 and in Lithuania – 0.0029. The allele frequencies of *CAPN3* gene mutation c.2288A > G and *DYSF* gene mutation c.4872delG are 0.003 in Latvia and profiled the known common mutations causing LGMD-2.

### Ethics approval and consent to participate

The study was performed with permission issued by the Central Medical Ethics Committee of Latvia (N27 from December 14, 2011). All participants or their parents signed an informed consent form to participate.

### Consent for publications

All participants or their parents signed an informed consent form to publish.

### Availability of data and materials

Microarray data are available in the ArrayExpress database (www.ebi.ac.uk/arrayexpress) under accession number E-MTAB-4662.

## Additional file

Additional file 1: Quality parameters and description of all mutations analyzed with Illumina VeraCode GoldenGate assay. (DOCX 69 kb)

## Abbreviations

LGMD: limb-girdle muscular dystrophy
CK: creatine kinase
EMG: electromyography
SNP: single nucleotide polymorphism
PCR: polymerase chain reaction
CI: confidence interval
*DYSF*: dysferlin
*FKRP*: fukutin related protein
*ANO5*: anoctamin 5
*CAPN3*: calpain 3
*SGCA*: sarcoglycan alpha
*SGCB*: sarcoglycan beta
*SGCD*: sarcoglycan delta
*SGCG*: sarcoglycan gamma
*CLCN1*: chloride voltage-gated channel 1
