# Supplementary material for: Robust genotyping tool for autosomal recessive type of limb-girdle muscular dystrophies
Source: BMC Musculoskelet Disord. 2016 May 4;17:200. doi: 10.1186/s12891-016-1058-z (PMC4855345; doi:10.1186/s12891-016-1058-z)
Supplement: Additional file 1: — Quality parameters and description of all mutations analyzed with Illumina VeraCode GoldenGate assay. (DOCX 69 kb) [file 12891_2016_1058_MOESM1_ESM.docx]

**Supplement Table S1**

**Quality parameters of Genome Studio analysis for all tested mutations**

| **Name** | **Position** | **Call Frequency** | **SNP** | **Gene Training Score** | **Original Score** | **Cluster Separa tion** | **AB T Mean** | **AB T Dev** | **AB R Mean** | **AB R Dev** |
| --- | --- | --- | --- | --- | --- | --- | --- | --- | --- | --- |
| **DYSF_00004** | **71909727** | **1** | **[A/G]** | **0,5646** | **0,6083** | **0,9292** | **0,1706** | **0,0122** | **0,7796** | **0,3096** |
| **DYSF_00053** | **7178022** | **1** | **[T/C]** | **0,6486** | **0,5872** | **0,6267** | **0,2005** | **0,0136** | **0,7747** | **0,1489** |
| rs56391414 | 135292087 | 1 | [T/C] | 0,4921 | 0,5194 | 0,5547 | 0,1339 | 0,0078 | 0,5533 | 0,1879 |
| CAPN3_00034 | 42693953 | 1 | [T/C] | 0,8634 | 0,8044 | 0,5105 | 0,535 | 0,0388 | 1,0584 | 0,1896 |
| **CAPN3_00289** | **42701997** | **1** | **[A/T]** | **0,4544** | **0,5926** | **0,4881** | **0,1911** | **0,0095** | **0,649** | **0,1039** |
| **DYSF_00173** | **71827961** | **1** | **[T/C]** | **0,5709** | **0,4335** | **0,4754** | **0,1787** | **0,0176** | **0,5973** | **0,2216** |
| DYSF_00305 | 7170918 | 1 | [A/G] | 0,4825 | 0,496 | 0,4724 | 0,8014 | 0,0067 | 1,0333 | 0,3653 |
| DYSF_00039 | 71753416 | 1 | [C/G] | 0,4921 | 0,5125 | 0,4666 | 0,8023 | 0,0135 | 0,9693 | 0,2024 |
| **DYSF_00233** | **71906322** | **1** | **[T/C]** | **0,8031** | **0,8031** | **0,4595** | **0,5418** | **0,0609** | **0,9835** | **0,0994** |
| rs2306942 | 129635800 | 1 | [T/C] | 0,7846 | 0,7624 | 0,4531 | 0,378 | 0,0498 | 0,708 | 0,3939 |
| **CAPN3_00129** | **42695076** | **1** | **[A/G]** | **0,7332** | **0,6697** | **0,4481** | **0,449** | **0,0354** | **1,0235** | **0,1415** |
| DYSF_00187 | 71730372 | 1 | [A/G] | 0,5076 | 0,5002 | 0,4433 | 0,1984 | 0,0145 | 0,9445 | 0,3335 |
| rs77138370 | 47259529 | 1 | [C/G] | 0,7321 | 0,6778 | 0,4387 | 0,5053 | 0,0272 | 0,9513 | 0,3212 |
| **DYSF_00016** | **71797014** | **1** | **[A/G]** | **0,7319** | **0,6733** | **0,4342** | **0,4431** | **0,0374** | **1,7263** | **0,2063** |
| CAPN3_00143 | 42652248 | 1 | [T/C] | 0,7893 | 0,7893 | 0,4275 | 0,5287 | 0,064 | 1,1168 | 0,0996 |
| **DYSF_00064** | **71740998** | **1** | **[T/C]** | **0,5949** | **0,4929** | **0,4154** | **0,1836** | **0,0147** | **0,8101** | **0,2079** |
| **CAPN3_00147** | **42681236** | **1** | **[T/G]** | **0,7966** | **0,7966** | **0,4118** | **0,4716** | **0,0684** | **1,1664** | **0,0558** |
| CAPN3_00430 | 42689074 | 1 | [A/G] | 0,7916 | 0,7916 | 0,408 | 0,4737 | 0,0684 | 1,2163 | 0,2772 |
| CAPN3_00125 | 42703969 | 1 | [A/G] | 0,7863 | 0,7863 | 0,4042 | 0,474 | 0,0694 | 1,2852 | 0,1274 |
| SGCB_00001 | 52895065 | 1 | [G/C] | 0,4402 | 0,5846 | 0,3985 | 0,1515 | 0,0054 | 0,5233 | 0,1505 |
| **DYSF_00014** | **71896321** | **1** | **[A/G]** | **0,4511** | **0,4866** | **0,3807** | **0,1393** | **0,0104** | **0,8184** | **0,1612** |
| CAPN3_00119 | 42703106 | 1 | [A/G] | 0,7689 | 0,7411 | 0,379 | 0,49 | 0,0546 | 1,3734 | 0,0921 |
| SGCG_00008 | 23894778 | 1 | [A/G] | 0,7777 | 0,7777 | 0,3766 | 0,4808 | 0,0733 | 1,0996 | 0,0659 |
| SGCA_00005 | 48247606 | 1 | [T/C] | 0,6865 | 0,6577 | 0,369 | 0,4634 | 0,049 | 0,9053 | 0,3491 |
| CAPN3_00382 | 42678468 | 1 | [D/I] | 0,3724 | 0,6449 | 0,3676 | 0,1454 | 0,0127 | 0,8292 | 0,2662 |
| **CAPN3_00104** | **42701567** | **1** | **[I/D]** | **0,5803** | **0,4198** | **0,3577** | **0,8019** | **0,0101** | **0,7972** | **0,0868** |
| **DYSF_00205** | **71742846** | **1** | **[T/C]** | **0,7024** | **0,7024** | **0,3441** | **0,5076** | **0,0732** | **1,2513** | **0,1144** |
| **CAPN3_00040** | **42700425** | **1** | **[T/C]** | **0,7107** | **0,6942** | **0,3399** | **0,4695** | **0,0634** | **1,4883** | **0,1585** |
| rs60890628 | 156108298 | 1 | [T/C] | 0,3964 | 0,4314 | 0,3153 | 0,1409 | 0,0116 | 0,7836 | 0,2439 |
| rs8142483 | 34000484 | 1 | [A/G] | 0,4658 | 0,4663 | 0,3122 | 0,152 | 0,0114 | 0,7503 | 0,2673 |
| SGCD_00003 | 155771584 | 1 | [T/C] | 0,6741 | 0,6378 | 0,2928 | 0,4273 | 0,0649 | 1,0489 | 0,1612 |
| **CAPN3_00118** | **42702630** | **1** | **[T/C]** | **0,6027** | **0,6623** | **0,2746** | **0,7122** | **0,0108** | **1,2935** | **0,1445** |
| DYSF_00007 | 71791204 | 1 | [C/G] | 0,4788 | 0,3241 | 0,2347 | 0,2899 | 0,0191 | 0,5701 | 0,1487 |
| **CAPN3_00222** | **42678383** | **0,9946** | **[A/G]** | **0,6397** | **0,786** | **0,5729** | **0,5594** | **0,0394** | **0,6697** | **0,05** |
| **CAPN3_00010** | **42680002** | **0,9946** | **[I/D]** | **0,5408** | **0,6539** | **0,5659** | **0,7755** | **0,0156** | **1,3593** | **0,5051** |
| SGCA_00001 | 48245078 | 0,9946 | [T/C] | 0,6148 | 0,4831 | 0,4603 | 0,1677 | 0,0098 | 0,8037 | 0,3162 |
| SGCB_00016 | 52896002 | 0,9946 | [A/G] | 0,8097 | 0,7547 | 0,4542 | 0,5249 | 0,0367 | 1,0217 | 0,0815 |
| rs36215895 | 64676751 | 0,9946 | [A/G] | 0,4201 | 0,2392 | 0,3664 | 0,1946 | 0,0188 | 0,6616 | 0,1375 |
| **CAPN3_00108** | **42702191** | **0,9946** | **[C/G]** | **0,3843** | **0,558** | **0,3516** | **0,8041** | **0,0113** | **0,7772** | **0,2377** |
| **DYSF_00207** | **71743373** | **0,9946** | **[D/I]** | **0,7026** | **0,7026** | **0,2765** | **0,4622** | **0,0886** | **0,9831** | **0,0974** |
| **CAPN3_00123** | **42702843** | **0,9891** | **[A/G]** | **0,7607** | **0,7463** | **0,3544** | **0,4896** | **0,0493** | **1,2144** | **0,1453** |
| CAPN3_00427 | 42703494 | 0,9891 | [T/G] | 0,3656 | 0,5801 | 0,3289 | 0,8797 | 0,009 | 0,6587 | 0,197 |
| rs2287717 | 47258842 | 0,9891 | [A/G] | 0,6507 | 0,3543 | 0,3069 | 0,1704 | 0,0196 | 0,8575 | 0,2335 |
| DYSF_00022 | 71788919 | 0,9891 | [D/I] | 0,474 | 0,3343 | 0,2972 | 0,1812 | 0,0215 | 0,7708 | 0,2872 |
| SGCA_00003 | 48245014 | 0,9891 | [A/G] | 0,4265 | 0,3084 | 0,2557 | 0,2431 | 0,0205 | 0,4386 | 0,139 |
| **DYSF_00043** | **71762412** | **0,9837** | **[A/C]** | **0,7124** | **0,7792** | **0,6912** | **0,4813** | **0,0344** | **0,7635** | **0,3103** |
| rs58932704 | 156106204 | 0,9837 | [A/G] | 0,7801 | 0,7801 | 0,3754 | 0,4993 | 0,0695 | 1,2574 | 0,2978 |
| SGCG_00055 | 23898591 | 0,9821 | [A/G] | 0,3353 | 0,5845 | 0,3163 | 0,13 | 0,0128 | 0,7795 | 0,275 |
| rs60458016 | 156105827 | 0,9783 | [A/G] | 0,7605 | 0,7444 | 0,3624 | 0,4786 | 0,0525 | 1,3273 | 0,1515 |
| DYSF_00236 | 71744158 | 0,9783 | [T/C] | 0,401 | 0,5401 | 0,3214 | 0,149 | 0,0121 | 0,6982 | 0,249 |
| rs916130 | 23898997 | 0,9783 | [T/C] | 0,5032 | 0,3166 | 0,2528 | 0,4856 | 0,142 | 0,58 | 0,248 |
| rs2070818 | 153609158 | 0,9728 | [C/G] | 0,4583 | 0,5995 | 0,4447 | 0,8373 | 0,0084 | 0,6966 | 0,1238 |
| SGCG_00002 | 23898652 | 0,962 | [A/G] | 0,4601 | 0,4428 | 0,3769 | 0,129 | 0,0151 | 0,7922 | 0,1886 |
| DYSF_00002 | 71896803 | 0,962 | [D/I] | 0,5001 | 0,6694 | 0,3129 | 0,1303 | 0,0117 | 1,0949 | 0,1089 |
| CAPN3_00212 | 42702694 | 0,962 | [A/G] | 0,3948 | 0,3255 | 0,3001 | 0,158 | 0,0136 | 0,8542 | 0,3449 |
| CAPN3_00298 | 42691698 | 0,9511 | [A/G] | 0,4112 | 0,6349 | 0,3461 | 0,8478 | 0,0083 | 0,8765 | 0,3316 |
| CAPN3_00001 | 42676699 | 0,9511 | [T/C] | 0,3375 | 0,6629 | 0,2589 | 0,1319 | 0,0088 | 0,9215 | 0,1013 |
| SGCA_00023 | 48245366 | 0,9457 | [T/C] | 0,6392 | 0,294 | 0,7125 | 0,8056 | 0,0091 | 0,9952 | 0,2386 |
| DYSF_00178 | 71887767 | 0,9402 | [D/I] | 0,4223 | 0,5778 | 0,4405 | 0,132 | 0,0151 | 0,7823 | 0,2446 |
| DYSF_00239 | 71838611 | 0,9348 | [T/C] | 0,5941 | 0,2274 | 0,6126 | 0,8022 | 0,0143 | 0,4086 | 0,0745 |
| SGCD_00001 | 156184673 | 0,9348 | [D/I] | 0,4235 | 0,5042 | 0,5073 | 0,1522 | 0,008 | 0,8723 | 0,2269 |
| DYSF_00001 | 71780201 | 0,9348 | [A/G] | 0,4974 | 0,4634 | 0,2525 | 0,1572 | 0,0136 | 0,8224 | 0,2751 |
| DYSF_00116 | 71747339 | 0,9293 | [A/G] | 0,3916 | 0,513 | 0,422 | 0,104 | 0,012 | 0,7845 | 0,2478 |
| DYSF_00139 | 71742780 | 0,9239 | [A/G] | 0,3682 | 0,2972 | 0,3295 | 0,112 | 0,0115 | 0,9154 | 0,2543 |
| rs34551924 | 6740704 | 0,913 | [T/C] | 0,7888 | 0,7888 | 0,4038 | 0,4745 | 0,0693 | 1,141 | 0,4764 |
| CAPN3_00193 | 42652143 | 0,8859 | [I/D] | 0,3544 | 0,3702 | 0,297 | 0,9508 | 0,0115 | 0,7426 | 0,2739 |
| rs946252 | 11313027 | 0,8696 | [T/C] | 0,7327 | 0,649 | 0,5507 | 0,5055 | 0,0785 | 1,0626 | 0,2013 |
| DYSF_00155 | 71838463 | 0,837 | [A/C] | 0,6623 | 0,652 | 0,7447 | 0,7119 | 0,0131 | 0,4953 | 0,095 |
| CAPN3_00027 | 42681157 | 0,8261 | [T/C] | 0,4553 | 0,5873 | 0,3509 | 0,1484 | 0,0082 | 0,4455 | 0,098 |
| SGCA_00084 | 48245923 | 0,8207 | [T/C] | 0,4975 | 0,3002 | 0,3168 | 0,138 | 0,0157 | 0,9643 | 0,3182 |
| SGCA_00009 | 48246607 | 0,788 | [A/G] | 0,765 | 0,7081 | 0,4218 | 0,4989 | 0,0289 | 0,7291 | 0,1196 |
| SGCB_00012 | 52895932 | 0,7772 | [A/G] | 0,7324 | 0,54 | 0,4284 | 0,5204 | 0,023 | 0,9017 | 0,1472 |
| CAPN3_00043 | 42695169 | 0,7337 | [T/C] | 0,3634 | 0,6602 | 0,3089 | 0,1061 | 0,0117 | 0,649 | 0,2196 |
| DYSF_00066 | 71795213 | 0,7283 | [T/C] | 0,3364 | 0,3685 | 0,3389 | 0,0849 | 0,0101 | 0,7135 | 0,2228 |
| CAPN3_00141 | 42693865 | 0,6413 | [A/G] | 0,5315 | 0,5375 | 0,3428 | 0,1208 | 0,0109 | 0,9008 | 0,3867 |
| SGCA_00034 | 48245758 | 0,6359 | [T/C] | 0,4932 | 0,6431 | 0,3081 | 0,1068 | 0,0164 | 0,8918 | 0,3718 |
| SGCA_00006 | 48244792 | 0,5598 | [A/G] | 0,3699 | 0,3555 | 0,2375 | 0,0639 | 0,0106 | 0,7472 | 0,2684 |
| CAPN3_00390 | 42682197 | 0 | [A/G] | 0 | 0,4025 | 0,3957 | 0,9174 | 0,0047 | 0,8221 | 0,0819 |
| DYSF_00126 | 71894607 | 0 | [A/G] | 0 | 0,4274 | 0,2329 | 0,6299 | 0,1381 | 0,6162 | 0,147 |
| DYSF_00131 | 7178214 | 0 | [G/C] | 0 | 0,422 | 0,2242 | 0,4413 | 0,1231 | 0,6586 | 0,0935 |
| DYSF_00246 | 71730438 | 0 | [A/G] | 0 | 0,5674 | 0,1937 | 0,4183 | 0,1594 | 0,9793 | 0,1051 |
| DYSF_00213 | 71795437 | 0 | [D/I] | 0 | 0,5722 | 0,1903 | 0,5125 | 0,1827 | 0,8434 | 0,0835 |
| CAPN3_00236 | 42684858 | 0 | [A/C] | 0 | 0,3467 | 0,1879 | 0,1109 | 0,003 | 0,2101 | 0,0374 |
| CAPN3_00096 | 42691839 | 0 | [T/C] | 0 | 0,4811 | 0,181 | 0,6722 | 0,1602 | 0,8853 | 0,1333 |
| SGCB_00014 | 52894973 | 0 | [A/G] | 0 | 0,5426 | 0,1809 | 0,5574 | 0,2175 | 0,5596 | 0,1572 |
| DYSF_00027 | 71886125 | 0 | [A/G] | 0 | 0,4796 | 0,1776 | 0,5744 | 0,1393 | 1,1815 | 0,0955 |
| SGCG_00001 | 23869573 | 0 | [I/D] | 0 | 0,6041 | 0,1742 | 0,453 | 0,2027 | 0,9852 | 0,1004 |
| DYSF_00360 | 71748001 | 0 | [T/G] | 0 | 0,4242 | 0,1725 | 0,4683 | 0,1714 | 0,4857 | 0,0589 |
| DYSF_00023 | 71797834 | 0 | [T/C] | 0 | 0,4877 | 0,1712 | 0,5729 | 0,1188 | 0,4652 | 0,1765 |
| CAPN3_00007 | 42686503 | 0 | [T/C] | 0 | 0,4951 | 0,1703 | 0,4644 | 0,1565 | 0,3529 | 0,1274 |
| DYSF_00203 | 71839797 | 0 | [T/G] | 0 | 0,485 | 0,1696 | 0,6992 | 0,1555 | 0,8542 | 0,0707 |
| DYSF_00117 | 71740897 | 0 | [T/G] | 0,2416 | 0,3963 | 0,1671 | 0,1383 | 0,0081 | 0,3983 | 0,0913 |
| rs75079578 | 47259292 | 0 | [T/C] | 0 | 0,2556 | 0,1555 | 0,4745 | 0,0855 | 1,1244 | 0,4599 |
| DYSF_00347 | 71783116 | 0 | [D/I] | 0 | 0,393 | 0,1491 | 0,6934 | 0,1551 | 0,8287 | 0,1361 |
| DYSF_00211 | 71778761 | 0 | [A/G] | 0 | 0,2667 | 0,1412 | 0,7568 | 0,1538 | 0,5558 | 0,0956 |
| SGCD_00006 | 155935695 | 0 | [A/C] | 0,2269 | 0,2269 | 0,1344 | 0,5964 | 0,064 | 0,5632 | 0,2694 |

Cluster separation, which measures the separation between the three genotype clusters; call frequency is the proportion of all samples at each locus with call scores above the no-call threshold; AB R Mean is the mean normalized intensity of the heterozygote cluster; AB T Mean is the mean normalized theta values of the heterozygote cluster.

**Supplement Table S2A.**

**The Group 1 mutations.**

| **Gene** | **Mutation ID** | **DNA change** | **Protein**  **change** | **Alleles reported** | **References** | **Geographic origin** |
| --- | --- | --- | --- | --- | --- | --- |
| *CAPN3* | CAPN3_00222 | 398C>T | Ala133Val | 2 | [1, 2, 3] | I |
| *CAPN3* | CAPN3_00010 | 550delA | Thr184Argfs*  36 | 225 | [4, 5, 2, 6] | Trans Europe, Turkey |
| *CAPN3* | CAPN3_00147 | 743T>G | Met248Arg | 4 | [7] | F, N, Brazil (Caucasian) |
| *CAPN3* | CAPN3_00129 | 1621C>T | Arg541Trp | 17 | [1, 2] | I, US, Hungary, Kurdish |
| *CAPN3* | CAPN3_00040 | 1817C>T | Ser606Leu | 10 | [5] | I, G, Portugal |
| *CAPN3* | CAPN3_00104 | 1981delA | Ile661* | 16 | [3, 8] | G, F, N, UK |
| *CAPN3* | CAPN3_00289 | 2005T>A | Cys669Ser | once | [9] | G |
| *CAPN3* | CAPN3_00108 | 2113G>C | Asp705His | 3 | [5] | F, G |
| *CAPN3* | CAPN3_00118 | 2120A>G | Asp707Gly | 14 | [10, 11] | Japan |
| *CAPN3* | CAPN3_00123 | 2242C>T | Arg748* | 39 | [1, 2, 12, 13, 14, 15] | I, G, N, Hungary, UK, Czech |
| *DYSF* | DYSF_00064 | 610C>T | Arg204* | 6 | [16, 17, 18] | G, F, US, Canada |
| *DYSF* | DYSF_00205 | 757C>T | Arg253Trp | 4 | [18, 16] | F, G, N |
| *DYSF* | DYSF_00207 | 855+1delG | p? | 12 | [18, 19, 16, 20, 21, 22] | G, F, UK, Switz. |
| *DYSF* | DYSF_00043 | 1368C>A | Cys456* | 3 | [9] | Mexico |
| *DYSF* | DYSF_00053 | 1834C>T | Gln612* | 4 | [23, 18, 19, 16, 17] | UK, US, Algeria |
| *DYSF* | DYSF_00016 | 2875C>T | Arg959Trp | 8 | [24, 25, 12, 26] | I |
| *DYSF* | DYSF_00173 | 3832C>T | Gln1278* | 4 | [18, 19, 16] | G, F, UK, US |
| *DYSF* | DYSF_00014 | 5509G>A | Asp1837Asn | 8 | [27, 24, 28, 16, 29] | I, S, F, US, Portugal, Japan |
| *DYSF* | DYSF_00233 | 5903G>A | Trp1968* | 4 | [18, 19, 16, 28] | F, S |
| *DYSF* | DYSF_00004 | 6124C>T | Arg2042Cys | 12 | [30, 31, 32, 24, 17, 12, 28, 19, 16, 33] | I, S, F, US, Canada, India |

I, Italy; F, France; G, Germany; S, Spain; N, Netherlands; Switz, Switzerland.

**Supplement Table S2B.**

**The Group 2 mutations.**

| **Gene** | **Mutation ID** | **DNA change** | **Protein change** | **Alleles reported** | **References** | **Geographic origin** |
| --- | --- | --- | --- | --- | --- | --- |
| *CAPN3* | CAPN3_00193 | 140_142del | Ile47del | 11 | [1, 6, 3] | I, S |
| *CAPN3* | CAPN3_00143 | 245C>T | Pro82Leu | 25 | [7, 1, 2] | I, S, G, US, Czech |
| *CAPN3* | CAPN3_00001 | 328C>T | Arg110* | 19 | [7] | I, G |
| *CAPN3* | CAPN3_00382 | 483delG | Ile162Serfs*17 | 2 | [3] | F |
| *CAPN3* | CAPN3_00027 | 664G>A | Gly222Arg | 19 | [34, 3] | S |
| *CAPN3* | CAPN3_00430 | 1192T>C | Trp398Arg | 4 | [12 | I |
| *CAPN3* | CAPN3_00298 | 1202A>G | Tyr401Cys | once | [9] | S |
| *CAPN3* | CAPN3_00141 | 1381C>T | Arg461Cys | 17 | [10, 11] | Japan |
| *CAPN3* | CAPN3_00034 | 1469G>A | Arg490Gln | 45 | [35, 2, 6, 36, 1] | I, F, S, US, UK, Turkey |
| *CAPN3* | CAPN3_00043 | 1714C>T | Arg572Trp | 14 | [2, 5, 3] | F, I |
| *CAPN3* | CAPN3_00212 | 2184G>A | p? | 7 | [1, 37] | I |
| *CAPN3* | CAPN3_00119 | 2288A>G | Tyr763Cys | 6 | [2, 38] | I, G, Portugal, US |
| *CAPN3* | CAPN3_00427 | 2390A>C | His797Pro | 4 | [12] | I |
| *CAPN3* | CAPN3_00125 | 2464T>C | *822Arg  ext62* | once | [9] |  |
| *DYSF* | DYSF_00305 | 154T>C | Trp52Arg | 2 | [28, 16] | S, F |
| *DYSF* | DYSF_00187 | 265C>T | Arg89* | 6 | [39, 16] | A, F, Algeria |
| *DYSF* | DYSF_00139 | 691C>T | Gln231* | 2 | [40, 41] | Japan, UK |
| *DYSF* | DYSF_00236 | 895G>A | Gly299Arg | 5 | [19, 16, 20, 21, 22] | G, F |
| *DYSF* | DYSF_00116 | 937+1G>A | p? | 7 | [41, 42, 43, 25, 16] | Japan, China |
| *DYSF* | DYSF_00039 | 1120G>C | Val374Leu | 8 | [44, 16, 25, 28, 12] | I, S, F, UK, Australia (Caucasian) |
| *DYSF* | DYSF_00001 | 1813C>T | Gln605* | 5 | [30, 31, 45, 21] | G, F |
| *DYSF* | DYSF_00022 | 2200_2204del | Thr734Pro  fs*18 | 5 | [25, 31, 12] | I |
| *DYSF* | DYSF_00007 | 2372C>G | Pro791Arg | 4 | [46, 31, 23, 47] | I, Canada (aboriginal) |
| *DYSF* | DYSF_00066 | 2643+1G>A | P? | 7 | [48, 29, 41, 16, 18, 19] | F, US, Portugal, Japan, Iran, Maghreb |
| *DYSF* | DYSF_00155 | 3992G>T | Arg1331Leu | 6 | [18, 24, 17] | I, F, UK, US |
| *DYSF* | DYSF_00239 | 4022T>C | Leu1341Pro | 2 | [20, 21, 40] | UK, Saudi Arabia |
| *DYSF* | DYSF_00178 | 4872delG | Glu1624Asp  fs*10 | 3 | [23, 26, 39] | I, Austria, Israel |
| *DYSF* | DYSF_00002 | 5594delG | Gly1865Ala  fs*101 | 12 | [18, 12, 16, 30, 31, 49] | I, F, S, Belgium, Portugal, Morocco |
| *SGCA* | SGCA_00003 | 229C>T | Arg77Cys | 50 | [34, 50, 51, 52, 53, 54, 55, 56, 57, 58, 59, 60, 61, 12] | S, G, I, F, N, US, Brazil, Japan |
| *SGCA* | SGCA_00023 | 371T>C | Ile124Thr | 15 | [55, 58, 62, 53, 61] | G, N, US, UK |
| *SGCA* | SGCA_00034 | 409G>A | Glu137Lys | 12 | [55, 63, 64, 60] | G, F, I, UK, Brazil |
| *SGCA* | SGCA_00084 | 574C>T | Arg192* | 7 | [9] | G, US |
| *SGCA* | SGCA_00009 | 739G>A | Val247Met | 30 | [54, 56, 65, 52, 12, 58] | G, F, I, N, UK, US, Brazil |
| *SGCA* | SGCA_00005 | 850C>T | Arg284Cys | 45 | [52, 50, 51, 61, 55, 12, 66] | F, I, Czech, US, Morocco, Brazil, Greece |
| *SGCB* | SGCB_00016 | 271C>T | Arg91Cys | 8 | [67, 12] | I, US (Amish) |
| *SGCB* | SGCB_00012 | 341C>T | Ser114Phe | 62 | [61, 12, 53, 65, 68] | G, F, I, N, UK, US, New Zealand |
| *SGCB* | SGCB_00001 | 452C>G | Thr151Arg | 8 | [67, 69] | US (Amish) |
| *SGCD* | SGCD_00003 | 89G>A | Trp30* | 3 | [61, 70] | US, India |
| *SGCD* | SGCD_00006 | 277G>A | Glu93* | 3 | [71, 70, 72, 52] | Turkey, India |
| *SGCD* | SGCD_00001 | 657delC | Thr220Pro  fs*6 | 8 | [52, 72] | Brazil |
| *SGCG* | SGCG_00001 | 525delT | Phe175Leu  fs*20 | 40 | [73, 74, 75, 52, 65, 60, 12] | G, I, N, India, Portugal, Israel, Brazil, Tunisia, Morocco, Australia |
| *SGCG* | SGCG_00008 | 581T>C | Leu194Ser | 10 | [70, 53] | N, UK, India |
| *SGCG* | SGCG_00055 | 787G>A | Glu263Lys | 10 | [76, 60] | G, N, US, UK |
| *SGCG* | SGCG_00002 | 848G>A | Cys283Tyr | 60 | [57, 12, 54, 77, 78, 79, 80, 81] | Gypsies all through the world |
| *LMNA* | rs60458016 | 1072G>A | Glu358Lys | 10 | [82, 83, 84] | F, UK, US |
| *LMNA* | rs58932704 | 1357C>T | Arg453Trp | 8 | [85, 86, 82] | I, S, UK, Poland |
| *LMNA* | rs60890628 | 1718C>T | Ser573Leu | 5 | [87, 88, 89] | I, US |
| *SYNE2* | rs36215895 | 18632C>T | Thr6211Met | MAF:T=0.005 (1000 genomes) | [90] |  |
| *AMELX* | rs946252 | 54+65T>C |  |  | [90] | gender control |
| *AMELY* | rs34551924 | -12-43T>C |  |  | [90] | gender control |
| *EMD* | rs2070818 | 445G>C | Asp149His | MAF:G=0.002 (1000 genomes) | [90] | polymorphism control |
| *FHL1* | rs56391414 | 794G>A | Cys265Tyr | MAF: NA | [90] | polymorphism control |
| *FKRP* | rs77138370 | 822C>G | Ile274Met | MAF:G=0.007 (1000 genomes) | [90] | polymorphism control |
| *FKRP* | rs2287717 | 135C>T | Ala45= | MAF:T=0.142 (1000 genomes) | [90] | polymorphism control |
| *LAMA2* | rs2306942 | 3412G>A | Val1138Met | MAF:A=0.106 (1000 genomes) | [90] | polymorphism control |
| *LARGE* | rs8142483 | 552G>A | Thr184= | MAF:T=0.009 (1000 genomes) | [90] | polymorphism control |
| *MYH7* | rs916130 | 1125A>G | Pro375= | MAF: NA | [90] | polymorphism control |

I, Italy; F, France; G, Germany; S, Spain; N, Netherlands; Switz, Switzerland.

**Supplement Table S2C.**

**Mutations, excluded from the LGMD-2 test kit.**

| **Gene** | **Mutation ID** | **DNA change** | **Protein change** |
| --- | --- | --- | --- |
| *CAPN3* | CAPN3_00390 | 848T>C | Mrt283Thr |
| *CAPN3* | CAPN3_00236 | 967G>T | Glu323* |
| *CAPN3* | CAPN3_00007 | 1079G>A | Trp360* |
| *CAPN3* | CAPN3_00096 | 1343G>A | Arg448His |
| *DYSF* | DYSF_00246 | 331C>T | Glu111* |
| *DYSF* | DYSF_00117 | 509C>A | Ala170Glu |
| *DYSF* | DYSF_00131 | 1566C>G | Tyr522* |
| *DYSF* | DYSF_00347 | 2077delC | His693Thrfs*4 |
| *DYSF* | DYSF_00213 | 2779delG | Ala927Leufs*2 |
| *DYSF* | DYSF_00023 | 3230G>A | Arg1046His |
| *DYSF* | DYSF_00173 | 3832C>T | Gln1278* |
| *DYSF* | DYSF_00027 | 4756C>T | Arg1607* |
| *DYSF* | DYSF_00126 | 5302C>T | Arg1789Trp |
| *DYSF* | DYSF_00203 | 4194C>A | Cys1398* |
| *DYSF* | DYSF_00360 | 1020C>A | Ser340Arg |
| *SGCA* | SGCA_00006 | 101G>A | Arg34His |
| *SGCA* | SGCA_00004 | 293G>A | Arg98His |
| *SGCB* | SGCB_00014 | 544A>G | Thr182Ala |
| *FKRP* | rs75079578 (polymorphism control) | 585C>T | Asp= |

[1] Piluso G, Politano L, Aurino S, Fanin M, Ricci E, Ventriglia VM, et al. Extensive scanning of the calpain-3 gene broadens the spectrum of LGMD2A phenotypes. J. Med. Genet. [Internet]. 2005 [cited 2016 Feb 25];42:686–93.

[2] Fanin M, Fulizio L, Nascimbeni AC, Spinazzi M, Piluso G, Ventriglia VM, et al. Molecular diagnosis in LGMD2A: mutation analysis or protein testing? Hum. Mutat. [Internet]. 2004 [cited 2016 Feb 25];24:52–62.

[3] Krahn M, Bernard R, Pecheux C, Hammouda EH, Eymard B, Lopez de Munain A, et al. Screening of the CAPN3 gene in patients with possible LGMD2A. Clin. Genet. [Internet]. 2006 [cited 2016 Feb 24];69:444–9.

[4] Dinçer P, Leturcq F, Richard I, Piccolo F, Yalnizoglu D, de Toma C, et al. A biochemical, genetic, and clinical survey of autosomal recessive limb girdle muscular dystrophies in Turkey. Ann. Neurol. [Internet]. 1997 [cited 2016 Feb 25];42:222–9.

[5] Richard I, Brenguier L, Dinçer P, Roudaut C, Bady B, Burgunder JM, et al. Multiple independent molecular etiology for limb-girdle muscular dystrophy type 2A patients from various geographical origins. Am. J. Hum. Genet. [Internet]. 1997 [cited 2016 Feb 25];60:1128–38.

[6] Fanin M, Nascimbeni AC, Fulizio L, Angelini C. The frequency of limb girdle muscular dystrophy 2A in northeastern Italy. Neuromuscul. Disord. [Internet]. 2005 [cited 2016 Feb 25];15:218–24.

[7] de Paula F, Vainzof M, Passos-Bueno MR, de Cássia M Pavanello R, Matioli SR, V B Anderson L, et al. Clinical variability in calpainopathy: what makes the difference? Eur. J. Hum. Genet. [Internet]. 2002 [cited 2016 Feb 25];10:825–32.

[8] Groen EJ, Charlton R, Barresi R, Anderson L V, Eagle M, Hudson J, et al. Analysis of the UK diagnostic strategy for limb girdle muscular dystrophy 2A. Brain [Internet]. 2007 [cited 2016 Feb 23];130:3237–49.

[9] http://www.dmd.nl/

[10] Minami N, Nishino I, Kobayashi O, Ikezoe K, Goto Y, Nonaka I. Mutations of calpain 3 gene in patients with sporadic limb-girdle muscular dystrophy in Japan. J. Neurol. Sci. [Internet]. 1999 [cited 2016 Feb 25];171:31–7.

[11] Chae J, Minami N, Jin Y, Nakagawa M, Murayama K, Igarashi F, et al. Calpain 3 gene mutations: genetic and clinico-pathologic findings in limb-girdle muscular dystrophy. Neuromuscul. Disord. [Internet]. 2001 [cited 2016 Feb 9];11:547–55.

[12] Guglieri M, Magri F, D’Angelo MG, Prelle A, Morandi L, Rodolico C, et al. Clinical, molecular, and protein correlations in a large sample of genetically diagnosed Italian limb girdle muscular dystrophy patients. Hum. Mutat. [Internet]. 2008 [cited 2016 Feb 25];29:258–66.

[13] Todorova A, Kress W, Mueller C. Novel mutations in the calpain 3 gene in Germany. Clin. Genet. [Internet]. 2005 [cited 2016 Feb 25];67:356–8.

[14] Chrobáková T, Hermanová M, Kroupová I, Vondrácek P, Maríková T, Mazanec R, et al. Mutations in Czech LGMD2A patients revealed by analysis of calpain3 mRNA and their phenotypic outcome. Neuromuscul. Disord. [Internet]. 2004 [cited 2016 Feb 25];14:659–65.

[15] Hermanová M, Zapletalová E, Sedlácková J, Chrobáková T, Letocha O, Kroupová I, et al. Analysis of histopathologic and molecular pathologic findings in Czech LGMD2A patients. Muscle Nerve [Internet]. 2006 [cited 2016 Feb 25];33:424–32.

[16] Krahn M, Béroud C, Labelle V, Nguyen K, Bernard R, Bassez G, et al. Analysis of the DYSF mutational spectrum in a large cohort of patients. Hum. Mutat. [Internet]. 2009 [cited 2016 Feb 25];30:E345–75.

[17] Kesari A, Fukuda M, Knoblach S, Bashir R, Nader GA, Rao D, et al. Dysferlin deficiency shows compensatory induction of Rab27A/Slp2a that may contribute to inflammatory onset. Am. J. Pathol. [Internet]. 2008 [cited 2016 Jan 27];173:1476–87.

[18] Nguyen K, Bassez G, Bernard R, Krahn M, Labelle V, Figarella-Branger D, et al. Dysferlin mutations in LGMD2B, Miyoshi myopathy, and atypical dysferlinopathies. Hum. Mutat. [Internet]. 2005 [cited 2012 Apr 17];26:165.

[19] Nguyen K, Bassez G, Krahn M, Bernard R, Laforêt P, Labelle V, et al. Phenotypic study in 40 patients with dysferlin gene mutations: high frequency of atypical phenotypes. Arch. Neurol. [Internet]. 2007 [cited 2016 Jan 29];64:1176–82.

[20] Wenzel K, Carl M, Perrot A, Zabojszcza J, Assadi M, Ebeling M, et al. Novel sequence variants in dysferlin-deficient muscular dystrophy leading to mRNA decay and possible C2-domain misfolding. Hum. Mutat. [Internet]. 2006 [cited 2016 Feb 25];27:599–600.

[21] Wenzel K, Geier C, Qadri F, Hubner N, Schulz H, Erdmann B, et al. Dysfunction of dysferlin-deficient hearts. J. Mol. Med. (Berl). [Internet]. 2007 [cited 2016 Feb 25];85:1203–14.

[22] Spuler S, Carl M, Zabojszcza J, Straub V, Bushby K, Moore SA, et al. Dysferlin-deficient muscular dystrophy features amyloidosis. Ann. Neurol. [Internet]. 2008 [cited 2016 Jan 27];63:323–8.

[23] Anderson L V, Harrison RM, Pogue R, Vafiadaki E, Pollitt C, Davison K, et al. Secondary reduction in calpain 3 expression in patients with limb girdle muscular dystrophy type 2B and Miyoshi myopathy (primary dysferlinopathies). Neuromuscul. Disord. [Internet]. 2000 [cited 2016 Feb 24];10:553–9.

[24] Cagliani R, Magri F, Toscano A, Merlini L, Fortunato F, Lamperti C, et al. Mutation finding in patients with dysferlin deficiency and role of the dysferlin interacting proteins annexin A1 and A2 in muscular dystrophies. Hum. Mutat. [Internet]. 2005 [cited 2016 Feb 25];26:283.

[25] Cagliani R, Fortunato F, Giorda R, Rodolico C, Bonaglia MC, Sironi M, et al. Molecular analysis of LGMD-2B and MM patients: identification of novel DYSF mutations and possible founder effect in the Italian population. Neuromuscul. Disord. [Internet]. 2003 [cited 2016 Feb 25];13:788–95.

[26] Kawabe K, Goto K, Nishino I, Angelini C, Hayashi YK. Dysferlin mutation analysis in a group of Italian patients with limb-girdle muscular dystrophy and Miyoshi myopathy. Eur. J. Neurol. [Internet]. 2004 [cited 2016 Feb 25];11:657–61.

[27] Ueyama H, Kumamoto T, Nagao S, Masuda T, Horinouchi H, Fujimoto S, et al. A new dysferlin gene mutation in two Japanese families with limb-girdle muscular dystrophy 2B and Miyoshi myopathy. Neuromuscul. Disord. [Internet]. 2001 [cited 2016 Feb 25];11:139–45.

[28] De Luna N, Freixas A, Gallano P, Caselles L, Rojas-García R, Paradas C, et al. Dysferlin expression in monocytes: a source of mRNA for mutation analysis. Neuromuscul. Disord. [Internet]. 2007 [cited 2016 Feb 25];17:69–76.

[29] Matsuda C, Hayashi YK, Ogawa M, Aoki M, Murayama K, Nishino I, et al. The sarcolemmal proteins dysferlin and caveolin-3 interact in skeletal muscle. Hum. Mol. Genet. [Internet]. 2001 [cited 2016 Feb 25];10:1761–6.

[30] Liu J, Aoki M, Illa I, Wu C, Fardeau M, Angelini C, et al. Dysferlin, a novel skeletal muscle gene, is mutated in Miyoshi myopathy and limb girdle muscular dystrophy. Nat. Genet. [Internet]. 1998 [cited 2016 Feb 25];20:31–6.

[31] Aoki M, Liu J, Richard I, Bashir R, Britton S, Keers SM, et al. Genomic organization of the dysferlin gene and novel mutations in Miyoshi myopathy. Neurology [Internet]. 2001 [cited 2016 Feb 25];57:271–8.

[32] Fanin M, Nascimbeni AC, Angelini C. Muscle protein analysis in the detection of heterozygotes for recessive limb girdle muscular dystrophy type 2B and 2E. Neuromuscul. Disord. [Internet]. 2006 [cited 2016 Feb 25];16:792–9.

[33] Khadilkar S V, Singh RK, Agarwal P, Krahn M, Levy N. Twenty-two year follow-up of an Indian family with dysferlinopathy-clinical, immunocytochemical, western blotting and genetic features. Neurol. India [Internet]. [cited 2016 Feb 25];56:388–90.

[34] Urtasun M, Sáenz A, Roudaut C, Poza JJ, Urtizberea JA, Cobo AM, et al. Limb-girdle muscular dystrophy in Guipúzcoa (Basque Country, Spain). Brain [Internet]. 1998 [cited 2016 Feb 25];121 ( Pt 9:1735–47.

[35] Fanin M, Nascimbeni AC, Fulizio L, Trevisan C Pietro, Meznaric-Petrusa M, Angelini C. Loss of calpain-3 autocatalytic activity in LGMD2A patients with normal protein expression. Am. J. Pathol. [Internet]. 2003 [cited 2016 Feb 9];163:1929–36.

[36] Fanin M, Nascimbeni AC, Angelini C. Screening of calpain-3 autolytic activity in LGMD muscle: a functional map of CAPN3 gene mutations. J. Med. Genet. [Internet]. 2007 [cited 2016 Feb 25];44:38–43.

[37] Sáenz A, Leturcq F, Cobo AM, Poza JJ, Ferrer X, Otaegui D, et al. LGMD2A: genotype-phenotype correlations based on a large mutational survey on the calpain 3 gene. Brain [Internet]. 2005 [cited 2016 Feb 8];128:732–42.

[38] Pizzanelli C, Mancuso M, Galli R, Choub A, Fanin M, Nascimbeni AC, et al. Epilepsy and limb girdle muscular dystrophy type 2A: double trouble, serendipitous finding or new phenotype? Neurol. Sci. [Internet]. 2006 [cited 2016 Feb 25];27:134–6.

[39] Walter MC, Braun C, Vorgerd M, Poppe M, Thirion C, Schmidt C, et al. Variable reduction of caveolin-3 in patients with LGMD2B/MM. J. Neurol. [Internet]. 2003 [cited 2016 Feb 25];250:1431–8.

[40] Klinge L, Aboumousa A, Eagle M, Hudson J, Sarkozy A, Vita G, et al. New aspects on patients affected by dysferlin deficient muscular dystrophy. J. Neurol. Neurosurg. Psychiatry [Internet]. 2010 [cited 2016 Feb 25];81:946–53.

[41] Takahashi T, Aoki M, Tateyama M, Kondo E, Mizuno T, Onodera Y, et al. Dysferlin mutations in Japanese Miyoshi myopathy: relationship to phenotype. Neurology [Internet]. 2003 [cited 2016 Feb 25];60:1799–804.

[42] Saito A, Higuchi I, Nakagawa M, Saito M, Hirata K, Suehara M, et al. Miyoshi myopathy patients with novel 5’ splicing donor site mutations showed different dysferlin immunostaining at the sarcolemma. Acta Neuropathol. [Internet]. 2002 [cited 2016 Feb 25];104:615–20.

[43] Ro L-S, Lee-Chen G-J, Lin T-C, Wu Y-R, Chen C-M, Lin C-Y, et al. Phenotypic features and genetic findings in 2 chinese families with Miyoshi distal myopathy. Arch. Neurol. [Internet]. 2004 [cited 2016 Feb 25];61:1594–9.

[44] Lo HP, Cooper ST, Evesson FJ, Seto JT, Chiotis M, Tay V, et al. Limb-girdle muscular dystrophy: diagnostic evaluation, frequency and clues to pathogenesis. Neuromuscul. Disord. [Internet]. 2008 [cited 2016 Feb 25];18:34–44.

[45] Diers A, Carl M, Stoltenburg-Didinger G, Vorgerd M, Spuler S. Painful enlargement of the calf muscles in limb girdle muscular dystrophy type 2B (LGMD2B) with a novel compound heterozygous mutation in DYSF. Neuromuscul. Disord. [Internet]. 2007 [cited 2016 Feb 25];17:157–62.

[46] Weiler T, Bashir R, Anderson L V, Davison K, Moss JA, Britton S, et al. Identical mutation in patients with limb girdle muscular dystrophy type 2B or Miyoshi myopathy suggests a role for modifier gene(s). Hum. Mol. Genet. [Internet]. 1999 [cited 2016 Feb 26];8:871–7.

[47] Therrien C, Dodig D, Karpati G, Sinnreich M. Mutation impact on dysferlin inferred from database analysis and computer-based structural predictions. J. Neurol. Sci. [Internet]. 2006 [cited 2016 Jan 27];250:71–8.

[48] Tagawa K, Ogawa M, Kawabe K, Yamanaka G, Matsumura T, Goto K, et al. Protein and gene analyses of dysferlinopathy in a large group of Japanese muscular dystrophy patients. J. Neurol. Sci. [Internet]. 2003 [cited 2016 Feb 26];211:23–8.

[49] Illa I, Serrano-Munuera C, Gallardo E, Lasa A, Rojas-García R, Palmer J, et al. Distal anterior compartment myopathy: a dysferlin mutation causing a new muscular dystrophy phenotype. Ann. Neurol. [Internet]. 2001 [cited 2016 Feb 26];49:130–4.

[50] Bueno MR, Moreira ES, Vainzof M, Chamberlain J, Marie SK, Pereira L, et al. A common missense mutation in the adhalin gene in three unrelated Brazilian families with a relatively mild form of autosomal recessive limb-girdle muscular dystrophy. Hum. Mol. Genet. [Internet]. 1995 [cited 2016 Feb 26];4:1163–7.

[51] Passos-Bueno MR, Moreira ES, Marie SK, Bashir R, Vasquez L, Love DR, et al. Main clinical features of the three mapped autosomal recessive limb-girdle muscular dystrophies and estimated proportion of each form in 13 Brazilian families. J. Med. Genet. [Internet]. 1996 [cited 2016 Feb 26];33:97–102.

[52] Moreira ES, Vainzof M, Suzuki OT, Pavanello RCM, Zatz M, Passos-Bueno MR. Genotype-phenotype correlations in 35 Brazilian families with sarcoglycanopathies including the description of three novel mutations. J. Med. Genet. [Internet]. 2003 [cited 2016 Feb 26];40:E12.

[53] Ginjaar HB, van der Kooi AJ, Ceelie H, Kneppers AL, van Meegen M, Barth PG, et al. Sarcoglycanopathies in Dutch patients with autosomal recessive limb girdle muscular dystrophy. J. Neurol. [Internet]. 2000 [cited 2016 Feb 26];247:524–9.

[54] Piccolo F, Roberds SL, Jeanpierre M, Leturcq F, Azibi K, Beldjord C, et al. Primary adhalinopathy: a common cause of autosomal recessive muscular dystrophy of variable severity. Nat. Genet. [Internet]. 1995 [cited 2016 Feb 26];10:243–5.

[55] Carrié A, Piccolo F, Leturcq F, de Toma C, Azibi K, Beldjord C, et al. Mutational diversity and hot spots in the alpha-sarcoglycan gene in autosomal recessive muscular dystrophy (LGMD2D). J. Med. Genet. [Internet]. 1997 [cited 2016 Feb 26];34:470–5.

[56] Eymard B, Romero NB, Leturcq F, Piccolo F, Carrié A, Jeanpierre M, et al. Primary adhalinopathy (alpha-sarcoglycanopathy): clinical, pathologic, and genetic correlation in 20 patients with autosomal recessive muscular dystrophy. Neurology [Internet]. 1997 [cited 2016 Feb 26];48:1227–34.

[57] Boito C, Fanin M, Siciliano G, Angelini C, Pegoraro E. Novel sarcoglycan gene mutations in a large cohort of Italian patients. J. Med. Genet. [Internet]. 2003 [cited 2016 Feb 26];40:e67.

[58] Klinge L, Dekomien G, Aboumousa A, Charlton R, Epplen JT, Barresi R, et al. Sarcoglycanopathies: can muscle immunoanalysis predict the genotype? Neuromuscul. Disord. [Internet]. 2008 [cited 2016 Feb 26];18:934–41.

[59] Hackman P, Juvonen V, Sarparanta J, Penttinen M, Aärimaa T, Uusitalo M, et al. Enrichment of the R77C alpha-sarcoglycan gene mutation in Finnish LGMD2D patients. Muscle Nerve [Internet]. 2005 [cited 2016 Feb 26];31:199–204.

[60] Trabelsi M, Kavian N, Daoud F, Commere V, Deburgrave N, Beugnet C, et al. Revised spectrum of mutations in sarcoglycanopathies. Eur. J. Hum. Genet. {EJHG} [Internet]. 2008 [cited 2012 Apr 17];16:793–803.

[61] Duggan DJ, Gorospe JR, Fanin M, Hoffman EP, Angelini C. Mutations in the sarcoglycan genes in patients with myopathy. N. Engl. J. Med. [Internet]. 1997 [cited 2012 Apr 17];336:618–24.

[62] Fischer D, Aurino S, Nigro V, Schröder R. On symptomatic heterozygous alpha-sarcoglycan gene mutation carriers. Ann. Neurol. [Internet]. 2003 [cited 2016 Feb 26];54:674–8.

[63] Gouveia TLF, Paim JFO, Pavanello RC, Zatz M, Vainzof M. Sarcoglycanopathies: a multiplex molecular analysis for the most common mutations. Diagn. Mol. Pathol. [Internet]. 2006 [cited 2016 Feb 26];15:95–100.

[64] Mongini T, Doriguzzi C, Bosone I, Chiadò-Piat L, Hoffman EP, Palmucci L. Alpha-sarcoglycan deficiency featuring exercise intolerance and myoglobinuria. Neuropediatrics [Internet]. 2002 [cited 2016 Feb 26];33:109–11.

[65] Crosbie RH, Lim LE, Moore SA, Hirano M, Hays AP, Maybaum SW, et al. Molecular and genetic characterization of sarcospan: insights into sarcoglycan-sarcospan interactions. Hum. Mol. Genet. [Internet]. 2000 [cited 2016 Feb 26];9:2019–27.

[66] Angelini C, Fanin M, Menegazzo E, Freda MP, Duggan DJ, Hoffman EP. Homozygous alpha-sarcoglycan mutation in two siblings: one asymptomatic and one steroid-responsive mild limb-girdle muscular dystrophy patient. Muscle Nerve [Internet]. 1998 [cited 2016 Feb 26];21:769–75.

[67] Duclos F, Broux O, Bourg N, Straub V, Feldman GL, Sunada Y, et al. Beta-sarcoglycan: genomic analysis and identification of a novel missense mutation in the LGMD2E Amish isolate. Neuromuscul. Disord. [Internet]. 1998 [cited 2016 Feb 26];8:30–8.

[68] Love DR. Limb girdle muscular dystrophy: use of dHPLC and direct sequencing to detect sarcoglycan gene mutations in a New Zealand cohort. Clin. Genet. [Internet]. 2004 [cited 2016 Feb 26];65:55–60.

[69] Lim LE, Duclos F, Broux O, Bourg N, Sunada Y, Allamand V, et al. Beta-sarcoglycan: characterization and role in limb-girdle muscular dystrophy linked to 4q12. Nat. Genet. [Internet]. 1995 [cited 2016 Feb 26];11:257–65.

[70] Khadilkar S V, Singh RK, Hegde M, Urtizberea A, Love DR, Chong B. Spectrum of mutations in sarcoglycan genes in the Mumbai region of western India: high prevalence of 525del T. Neurol. India [Internet]. [cited 2016 Feb 26];57:406–10.

[71] Dinçer P, Bönnemann CG, Erdir Aker O, Akçoren Z, Nigro V, Kunkel LM, et al. A homozygous nonsense mutation in delta-sarcoglycan exon 3 in a case of LGMD2F. Neuromuscul. Disord. [Internet]. 2000 [cited 2016 Feb 26];10:247–50.

[72] Nigro V, de Sá Moreira E, Piluso G, Vainzof M, Belsito A, Politano L, et al. Autosomal recessive limb-girdle muscular dystrophy, LGMD2F, is caused by a mutation in the delta-sarcoglycan gene. Nat. Genet. [Internet]. 1996 [cited 2016 Feb 26];14:195–8.

[73] McNally EM, Passos-Bueno MR, Bönnemann CG, Vainzof M, de Sá Moreira E, Lidov HG, et al. Mild and severe muscular dystrophy caused by a single gamma-sarcoglycan mutation. Am. J. Hum. Genet. [Internet]. 1996 [cited 2016 Feb 26];59:1040–7.

[74] Ben Othmane K, Ben Hamida M, Pericak-Vance MA, Ben Hamida C, Blel S, Carter SC, et al. Linkage of Tunisian autosomal recessive Duchenne-like muscular dystrophy to the pericentromeric region of chromosome 13q. Nat. Genet. [Internet]. 1992 [cited 2016 Feb 26];2:315–7.

[75] Dos Santos MR, Vieira EM, Reis Lima M. Molecular diagnosis and counseling in a family presenting compound heterozygosity for autosomal recessive limb-girdle muscular dystrophy. Genet. Couns. [Internet]. 2001 [cited 2016 Feb 26];12:223–9.

[76] Duncan DR, Kang PB, Rabbat JC, Briggs CE, Lidov HGW, Darras BT, et al. A novel mutation in two families with limb-girdle muscular dystrophy type 2C. Neurology [Internet]. 2006 [cited 2016 Feb 26];67:167–9.

[77] Todorova A, Ashikov A, Beltcheva O, Tournev I, Kremensky I. C283Y mutation and other C-terminal nucleotide changes in the gamma-sarcoglycan gene in the Bulgarian Gypsy population. Hum. Mutat. [Internet]. 1999 [cited 2016 Feb 26];14:40–4.

[78] Todorova A, Georgieva B, Tournev I, Todorov T, Bogdanova N, Mitev V, et al. A large deletion and novel point mutations in the calpain 3 gene (CAPN3) in Bulgarian LGMD2A patients. Neurogenetics [Internet]. 2007 [cited 2016 Feb 26];8:225–9.

[79] Lasa A, Piccolo F, de Diego C, Jeanpierre M, Colomer J, Rodríguez MJ, et al. Severe limb girdle muscular dystrophy in Spanish gypsies: further evidence for a founder mutation in the gamma-sarcoglycan gene. Eur. J. Hum. Genet. [Internet]. [cited 2016 Feb 26];6:396–9.

[80] García-García D, Teijeira-Bautista S, Fernández-Rodríguez JM, Flores-Calvete J, Sánchez-Espíldora P, Fernández-Couto D, et al. Gamma-sarcoglycanopathy: clinico-pathological and genetic study of 11 cases. Rev. Neurol. [Internet]. 1998 [cited 2016 Feb 26];26:905–11.

[81] Morar B, Gresham D, Angelicheva D, Tournev I, Gooding R, Guergueltcheva V, et al. Mutation history of the roma/gypsies. Am. J. Hum. Genet. [Internet]. 2004 [cited 2016 Feb 26];75:596–609.

[82] Bonne G, Mercuri E, Muchir A, Urtizberea A, Bécane HM, Recan D, et al. Clinical and molecular genetic spectrum of autosomal dominant Emery-Dreifuss muscular dystrophy due to mutations of the lamin A/C gene. Ann. Neurol. [Internet]. 2000 [cited 2016 Feb 26];48:170–80.

[83] Mercuri E, Poppe M, Quinlivan R, Messina S, Kinali M, Demay L, et al. Extreme variability of phenotype in patients with an identical missense mutation in the lamin A/C gene: from congenital onset with severe phenotype to milder classic Emery-Dreifuss variant. Arch. Neurol. [Internet]. 2004 [cited 2016 Feb 26];61:690–4.

[84] Quijano-Roy S, Mbieleu B, Bönnemann CG, Jeannet P-Y, Colomer J, Clarke NF, et al. De novo LMNA mutations cause a new form of congenital muscular dystrophy. Ann. Neurol. [Internet]. 2008 [cited 2016 Feb 26];64:177–86.

[85] Raffaele Di Barletta M, Ricci E, Galluzzi G, Tonali P, Mora M, Morandi L, et al. Different mutations in the LMNA gene cause autosomal dominant and autosomal recessive Emery-Dreifuss muscular dystrophy. Am. J. Hum. Genet. [Internet]. 2000 [cited 2016 Feb 26];66:1407–12.

[86] Vytopil M, Benedetti S, Ricci E, Galluzzi G, Dello Russo A, Merlini L, et al. Mutation analysis of the lamin A/C gene (LMNA) among patients with different cardiomuscular phenotypes. J. Med. Genet. [Internet]. 2003 [cited 2016 Jan 28];40:e132.

[87] Van Esch H, Agarwal AK, Debeer P, Fryns J-P, Garg A. A homozygous mutation in the lamin A/C gene associated with a novel syndrome of arthropathy, tendinous calcinosis, and progeroid features. J. Clin. Endocrinol. Metab. [Internet]. 2006 [cited 2016 Feb 26];91:517–21.

[88] Taylor MRG, Fain PR, Sinagra G, Robinson ML, Robertson AD, Carniel E, et al. Natural history of dilated cardiomyopathy due to lamin A/C gene mutations. J. Am. Coll. Cardiol. [Internet]. 2003 [cited 2016 Feb 26];41:771–80.

[89] Lanktree M, Cao H, Rabkin SW, Hanna A, Hegele RA. Novel LMNA mutations seen in patients with familial partial lipodystrophy subtype 2 (FPLD2; MIM 151660). Clin. Genet. [Internet]. 2007 [cited 2016 Feb 24];71:183–6.

[90] NCBI SNP database.
